# Tofacitinib Is an Effective Treatment for Refractory Scleromyositis Associated With Anti-PM/Scl

**DOI:** 10.7759/cureus.34125

**Published:** 2023-01-24

**Authors:** Jorge Álvarez Troncoso, Almudena Nuño González, Elena Martínez Robles, Raquel Sorriguieta Torre, Ángel Robles Marhuenda

**Affiliations:** 1 Internal Medicine, Systemic Autoimmune Diseases Unit, Hospital Universitario La Paz, Madrid, ESP; 2 Dermatology Department, Hospital Universitario La Paz, Madrid, ESP

**Keywords:** connective tissue disease associated interstitial lung disease, myositis, scleroderma, myocarditis, tofacitinib, calcinosis, scleromyositis

## Abstract

Scleromyositis is a rare autoimmune disease characterized by overlapping scleroderma and myositis. This case report discusses the presentation and management of a 28-year-old male with scleromyositis presenting with myositis, arthritis, Raynaud’s phenomenon, refractory calcinosis, interstitial lung disease, and myocarditis. This case highlights key points in the systematic approach to immunosuppressive treatment and proposes a novel therapeutic option.

## Introduction

Anti-PM/Scl-positive patients are characterized by an overlap between myositis and systemic sclerosis (SSc) [1]. Anti-PM/Scl are myositis-associated autoantibodies that define a characteristic phenotype with significant extra muscular involvement [1,2]. In addition to muscle involvement, interstitial lung disease, Raynaud's phenomenon, arthritis, mechanic's hands, sclerodactyly, telangiectasias, gastroesophageal reflux, subcutaneous edema, and calcinosis are common. There are reported cases of myocarditis also associated with this entity [1,2]. There is no clear consensus regarding the treatment of anti-PM/Scl, but there are case reports of the effectiveness of corticosteroids in combination with first-line classic immunosuppressive agents (azathioprine, methotrexate, mycophenolate) and second-line treatment with intravenous immunoglobulins (IVIg), or rituximab [1,2].

On the other hand, calcinosis is a manifestation present in systemic sclerosis and myositis (especially in juvenile dermatomyositis), refractory to standard classical and biological immunosuppressive treatments [3].

## Case presentation

**Figure 1:**
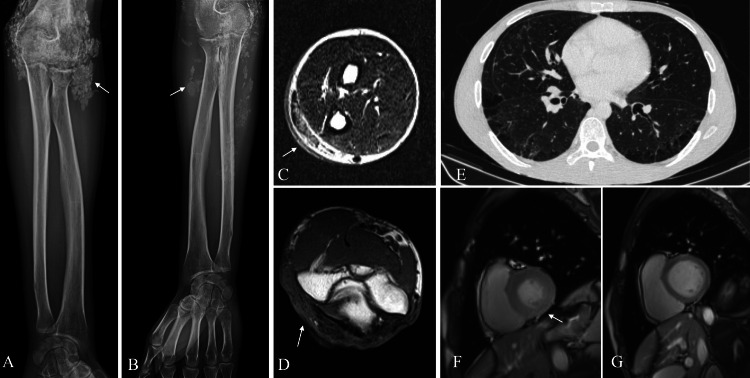
Systemic involvement of scleromyositis *A and B*: extensive calcinosis of the left and right elbow (arrows). *C*: MRI showing signs of cutaneous thickening with hypersignal of trabeculated aspect in long TR sequences (arrow). No involvement of muscle or bone planes. Small calcinosis plaques in dermis. *D:* Signal alteration of the subcutaneous cellular tissue in the form of hyposignal T1 compatible with cellulitis (arrow). Small calcinosis plaques in dermis.* E*: High-resolution CT scan showing bilateral pleuroapical thickening and paraseptal emphysema predominantly in the lower lobes and isolated areas of ground glass. *F*: Cardiac MRI 2021, diffuse myocardial inflammation with elevated T1 and T2 mapping, normal STIR with no evidence of fibrosis (arrow). *G*: Cardiac MRI 2022, no pathologic enhancements or evidence of inflammation or diffuse fibrosis.

**Figure 2:**
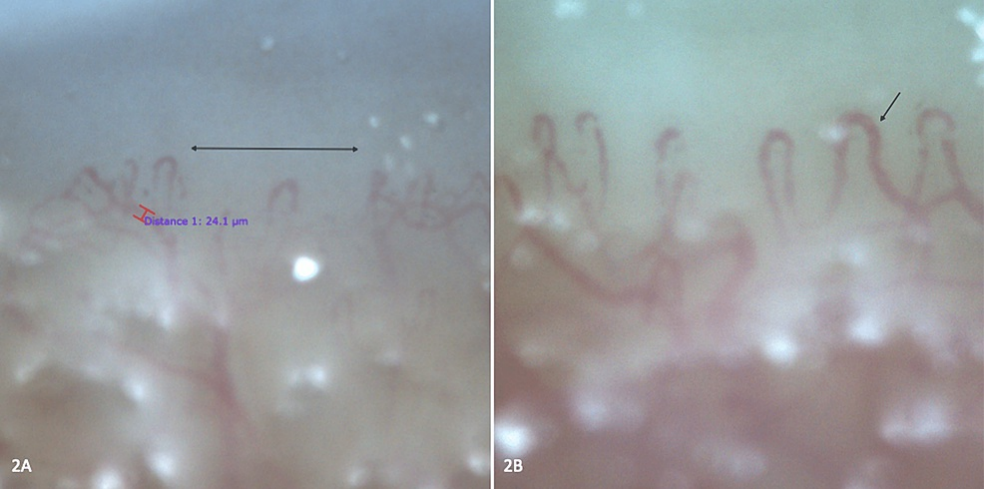
Nailfold capillaroscopy (200x) evolution *2A: *Nailfold capillaroscopy 2020: capillary loss with avascular areas (line), frequent branching, and arborizations without dilatations, hemorrhages, or megacapillaries. *2B*: Nailfold capillaroscopy 2022: partial recovery of the capillary bed but with neoangiogenesis pattern, dilatations (arrow), and arborescent branching without hemorrhages.

We present the case of a 28-year-old male with anti-PM/Scl-positive scleromyositis diagnosed in 2019. At disease onset, he presented with symmetrical proximal myositis predominantly in lower limbs, with CPK elevation (7891 UI/L) and confirmation by electromyogram, muscle MRI, and muscle biopsy (histopathology compatible with polymyositis). He associated telangiectasias, calcinosis, periungual and skin thickening (modified Rodnan skin score 4/52), and arthritis predominantly in the wrists, metacarpophalangeal and proximal interphalangeal joints. Besides, the erythrocyte sedimentation rate (ESR) was markedly elevated (49 mm/h). High-resolution pulmonary CT revealed interstitial lung disease with paraseptal emphysema and ground glass (Figure 1). Pulmonary function tests (PFTs) at diagnosis showed FVC 80%, FEV1 84%, FEV1/FVC 105%, DLCO 46%, and TLC 75% of the predicted value. He had Raynaud's phenomenon with numerous avascular areas, frequent branching, and arborization in nailfold capillaroscopy (Figure 2).

**Figure 3:**
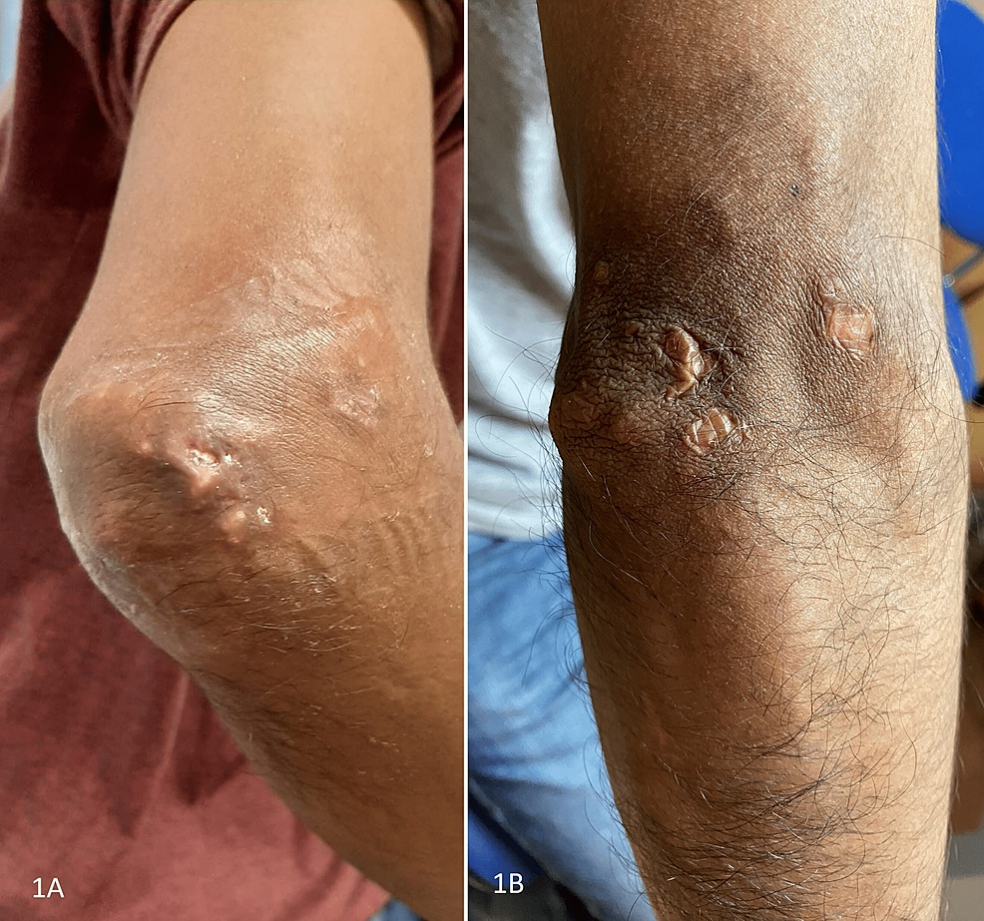
Improvement of calcinosis after treatment *1A*: Before tofacitinib. *1B*: After tofacitinib.

Given these data, he started treatment with steroids, mycophenolate mofetil (MMF), and IVIg, improving the muscular (partial), articular and pulmonary symptoms. Although these symptoms were controlled, the patient was still suffering from symptomatic myositis and refractory calcinosis; we tried first topical 25% thiosulfate cream, but there were no clinical or radiological results, so we tried intralesional thiosulfate (same concentration as the intravenous one) [4]. However, due to the refractoriness, rituximab (RTX) was added as induction and maintenance for one year. Despite all these treatments, in the year 2021, he presented a progression of calcinosis (Figure 3) in the shoulders, elbows, and thighs, requiring several local cures for skin exposure by the Dermatology Department and hospital admission for skin and soft tissue infection associated with calcinosis of the elbow. In addition, repeated laboratory tests showed mild elevation of ultrasensitive troponin (usTnI) (78.2 ng/L) and NTproBNP (124 pg/mL) associated with atypical chest pain without ECG or echocardiographic abnormalities. Cardiac MRI showed low-grade diffuse myocardial inflammation (in T1 and T2 mapping, with normal STIR) without areas of regional fibrosis compatible with myocarditis (Figure 1). 

Therefore, given the clinical severity and therapeutic refractoriness (steroids, MMF, IVIg, and RTX), tofacitinib 5 mg twice daily (previously, he received herpes zoster vaccination) was initiated in September 2021. From the initiation of tofacitinib until the present time (November 2022), the patient has not presented new hospital admissions, infections, soft tissue exposure, or progression of calcinosis. A clinical decrease in the extent of calcinosis has been observed, especially at both elbows (Figure 3). Arthritis and myositis are under control with normalization of ESR and CPK (3 mm/h and 86 UI/L, respectively) without myalgia or weakness (grade 5 on the Medical Research Council Scale for Muscle Strength). Pulmonary involvement is radiologically stable without ground glass infiltrates, and improvement in PFTs (FVC 90%, FEV1 90%, FEV1/FVC 100%, DLCO 56%, and TLC 91% of the predicted value).

Control nailfold capillaroscopy (Figure 2) showed improvement of the avascular areas without hemorrhages and persistence of branching and arborization of capillaries. Regarding myocarditis, usTnI and NTproBNP normalized (33.7 ng/L and <35 pg/mL, respectively) 3-4 months after the start of tofacitinib, and cardiac MRI at one year evidenced resolution of inflammatory infiltrates without residual fibrosis (Figure 1). Regarding treatment, tofacitinib was well-tolerated without safety issues and allowed progressive withdrawal of steroids up to prednisone 2.5 mg/24h at present. 

## Discussion

Anti-PM/Scl autoantibodies are associated with a mixed phenotype, including clinical features classically associated with idiopathic inflammatory myopathies and systemic sclerosis [1,2]. We highlight that despite severe manifestations, the response with tofacitinib is favorable after refractoriness to previously known therapies.

Tofacitinib is a JAK inhibitor approved for use in rheumatoid arthritis, psoriatic arthritis, ankylosing spondylitis, and ulcerative colitis. Tofacitinib reversibly inhibits JAK1 and JAK3 in vitro and, to a lesser extent, inhibits JAK2 and TYK2 [5]. TGF-beta signaling plays a central role in the pathogenesis of SSc. Thus, it is possible that JAK inhibitors can suppress TGF-beta signaling in the fibroblasts of SSc patients [6]. Also, JAK inhibitors could abrogate the proinflammatory profibrotic effects of T cells in vitro [7].

Recently, the experience of tofacitinib in case series of patients with refractory dermatomyositis [7-9], scleroderma [10], and calcinosis [11] has been published, demonstrating its usefulness as a steroid-sparing agent in the improvement of cutaneous, joint, muscle involvement and in CXCL9/CXCL10 and STAT1 biomarkers [7-11]. In addition to changes in biomarkers, recent studies demonstrate improvement in standardized clinical muscle (2016 ACR/EULAR myositis response criteria) and skin (CDASI activity score) scores within 12 weeks of treatment in patients with treatment-refractory active dermatomyositis [9]. There is evidence that the JAK/STAT signaling pathway is markedly activated in SSc patients and that tofacitinib significantly reduces the Rodnan score compared to methotrexate in patients with SSc [10]. In addition, there are also some publications on the usefulness of tofacitinib in the treatment of immune checkpoint inhibitor-associated cortico-resistant myocarditis [12], emphasizing its systemic utility beyond an exclusive organic indication. 

However, to our knowledge, there are no reported cases of anti-PM/Scl scleromyositis treated with tofacitinib, nor of its usefulness in treating both calcinosis and myocarditis associated with inflammatory myopathy.

## Conclusions

Anti-PM/Scl-associated scleromyositis is a systemic autoimmune disease characterized by an overlap of scleroderma and myositis that can lead to life-threatening disease. Myositis, Raynaud's phenomenon, skin lesions, and calcinosis are frequent and occasionally refractory. However, interstitial lung disease and myocardial involvement are less frequent but more severe. 

Treatment with classical immunosuppressants and biologics such as rituximab can be helpful in the management of the disease, but occasionally the response is insufficient. Tofacitinib is a promising therapy for scleromyositis, with emphasis on severe skin manifestations, myocarditis, and myositis refractory to classical and biological immunosuppressants.

## References

[1] De Lorenzo R, Pinal-Fernandez I, Huang W (2018). Muscular and extramuscular clinical features of patients with anti-PM/Scl autoantibodies. Neurology.

[2] Lazzaroni MG, Marasco E, Campochiaro C (2021). The clinical phenotype of systemic sclerosis patients with anti-PM/Scl antibodies: results from the EUSTAR cohort. Rheumatology (Oxford).

[3] Richardson C, Plaas A, Varga J (2020). Calcinosis in systemic sclerosis: Updates in pathophysiology, evaluation, and treatment. Curr Rheumatol Rep.

[4] Ma JE, Ernste FC, Davis MD, Wetter DA (2019). Topical sodium thiosulfate for calcinosis cutis associated with autoimmune connective tissue diseases: the Mayo Clinic experience, 2012-2017. Clin Exp Dermatol.

[5] McInnes IB, Byers NL, Higgs RE (2019). Comparison of baricitinib, upadacitinib, and tofacitinib mediated regulation of cytokine signaling in human leukocyte subpopulations. Arthritis Res Ther.

[6] Tang LY, Heller M, Meng Z, Yu LR, Tang Y, Zhou M, Zhang YE (2017). Transforming growth factor-β (TGF-β) directly activates the JAK1-STAT3 axis to induce hepatic fibrosis in coordination with the SMAD pathway. J Biol Chem.

[7] Chen Z, Wang X, Ye S (2019). Tofacitinib in amyopathic dermatomyositis-associated interstitial lung disease. N Engl J Med.

[8] Liu Y, Fang L, Chen W (2020). Identification of characteristics of overt myocarditis in adult patients with idiopathic inflammatory myopathies. Cardiovasc Diagn Ther.

[9] Paik JJ, Casciola-Rosen L, Shin JY (2021). Study of tofacitinib in refractory dermatomyositis: An open‐label pilot study of ten patients. Arthritis Rheumatol.

[10] Karalilova RV, Batalov ZA, Sapundzhieva TL, Matucci-Cerinic M, Batalov AZ (2021). Tofacitinib in the treatment of skin and musculoskeletal involvement in patients with systemic sclerosis, evaluated by ultrasound. Rheumatol Int.

[11] Wendel S, Venhoff N, Frye BC (2019). Successful treatment of extensive calcifications and acute pulmonary involvement in dermatomyositis with the Janus-Kinase inhibitor tofacitinib - A report of two cases. J Autoimmun.

[12] Wang C, Lin J, Wang Y (2021). Case series of steroid-resistant immune checkpoint inhibitor associated myocarditis: A comparative analysis of corticosteroid and tofacitinib treatment. Front Pharmacol.

